# Fournier’s Gangrene in an HIV-Positive Patient on Empagliflozin for the Treatment of Diabetes Mellitus

**DOI:** 10.7759/cureus.26083

**Published:** 2022-06-19

**Authors:** Farzam Khokhar, Carina Hernandez, Rahul Mahapatra

**Affiliations:** 1 Internal Medicine, State University of New York Upstate Medical University, Syracuse, USA; 2 Infectious Diseases, State University of New York Upstate Medical University, Syracuse, USA

**Keywords:** unusual etiology of fournier’s gangrene, pyogenic skin infection, types 2 diabetes, jardiance, empagliflozin, hiv aids, fournier’s gangrene in an immunocompetent patient

## Abstract

Fournier's gangrene is a severe polymicrobial infection that results in necrosis of the perineal and genital fasciae with rapid progression. This case report describes a 55-year-old male with a past medical history of HIV, type 2 diabetes, and hypertension who was diagnosed with Fournier's gangrene after the administration of empagliflozin (Jardiance). The patient presented with a worsening ulcer of the right groin and was diagnosed with Fournier’s gangrene based on clinical and radiographic findings. He underwent surgical debridement of the wound. The patient was treated with empiric vancomycin, piperacillin-tazobactam, and clindamycin. Wound cultures grew *Streptococcus anginosus* and *Staphylococcus epidermidis*. His antibiotic regimen was simplified to ampicillin-sulbactam. The patient required reconstructive surgery for wound closure after debridement. He received an additional 18 days of augmentin therapy with the resolution of the infectious process. At the time of Fournier’s gangrene onset, the patient's last HbA1C level was 8.2%, despite treatment with glipizide and empagliflozin. This case suggests an association between empagliflozin and Fournier’s gangrene in the setting of active HIV infection.

## Introduction

Fournier's gangrene is a rare but serious rapidly progressing necrotic infection of the scrotum, penis, or perineum. This leads to gangrene of the overlying dermal tissues, which rapidly spreads along the fascial planes. Fournier’s gangrene most commonly occurs due to a local infection adjacent to a point of entry such as an abscess, anal fissure, and colonic perforation. Urologic sources of infections include urinary tract infections, indwelling catheters, and neurogenic bladder. Other risk factors include HIV infection, renal failure, malignancy, obesity, localized trauma, smoking, and liver failure. Concomitant diabetes mellitus is found in over half of the patients with Fournier’s gangrene [1]. Sodium-glucose cotransporter-2 (SGLT-2) inhibitors are a class of drugs used to treat type 2 diabetes mellitus and work by enhancing glucosuria. The mechanism of action involves the inhibition of SGLT-2 in the proximal convoluted tubule to prevent reabsorption of glucose causing excretion of glucose in the urine. This enhanced glucosuria may increase the risk for urinary tract and genital infections.

## Case presentation

A 55-year-old man with a past medical history significant for HIV on highly active antiretroviral therapy (HAART), obstructive sleep apnea on continuous positive airway pressure (CPAP), type 2 diabetes mellitus, hyperlipidemia, and hypertension presented to the hospital with an ulcer in the right inguinal area that had worsened over the course of one week. The lesions had spread to the groin area and involved his right testicle. The pain had affected his sleep and the patient was having trouble ambulating due to testicular discomfort; he reported no difficulty in urinating. The patient was advised to present to the emergency department by his primary care physician.

For the management of his diabetes mellitus, the patient had been prescribed empagliflozin 12.5 mg daily as adjuvant therapy beginning 88 days prior to the admission. He had previously been on metformin, which had been discontinued due to gastrointestinal side effects. He had a significant past medical history of well-controlled HIV infection on Symtuza (darunavir 800 mg/cobicistat 150 mg/emtricitabine 200 mg/tenofovir alafenamide 10 mg) single pill formulation, which he had been on for several years and had tolerated well.

On admission, his vitals were stable - temperature: 98.3 °F, blood pressure: 131/84 mmHg, respiratory rate: 18 breaths per minute, and heart rate: 98 beats per minute. The patient's last HbA1c from approximately two months prior to presentation was 8.2%. The physical exam was pertinent for several furuncles noted in the right inguinal area. It had begun as a single lesion and progressed to three separate ulcerative lesions. Erythema, induration, and edema were also noted spreading to the scrotum.

**Figure 1:**
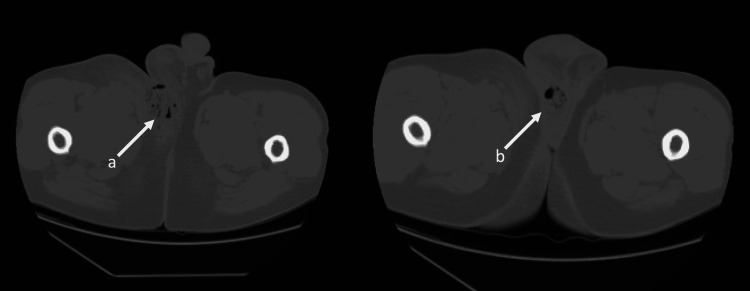
CT scan of the abdomen and pelvis The image shows marked inflammatory stranding and foci of air within the right groin, extending into the medial proximal right thigh and right scrotal wall (a). There was more localized fluid and air in the posterior right hemiscrotal wall (b) CT: computed tomography

Labs were notable for elevated white blood cell count at 13 × 10^9^/L and the lactic acid at 1.4. Hemoglobin and hematocrit were stable at 15.1 g/dl and 43.1% respectively. Creatinine was normal at 0.9 g/dl. A CT scan of the abdomen and pelvis with intravenous contrast showed marked inflammatory stranding and foci of air within the right groin extending into the medial proximal right thigh and right scrotal wall (Figure 1).

**Figure 2:**
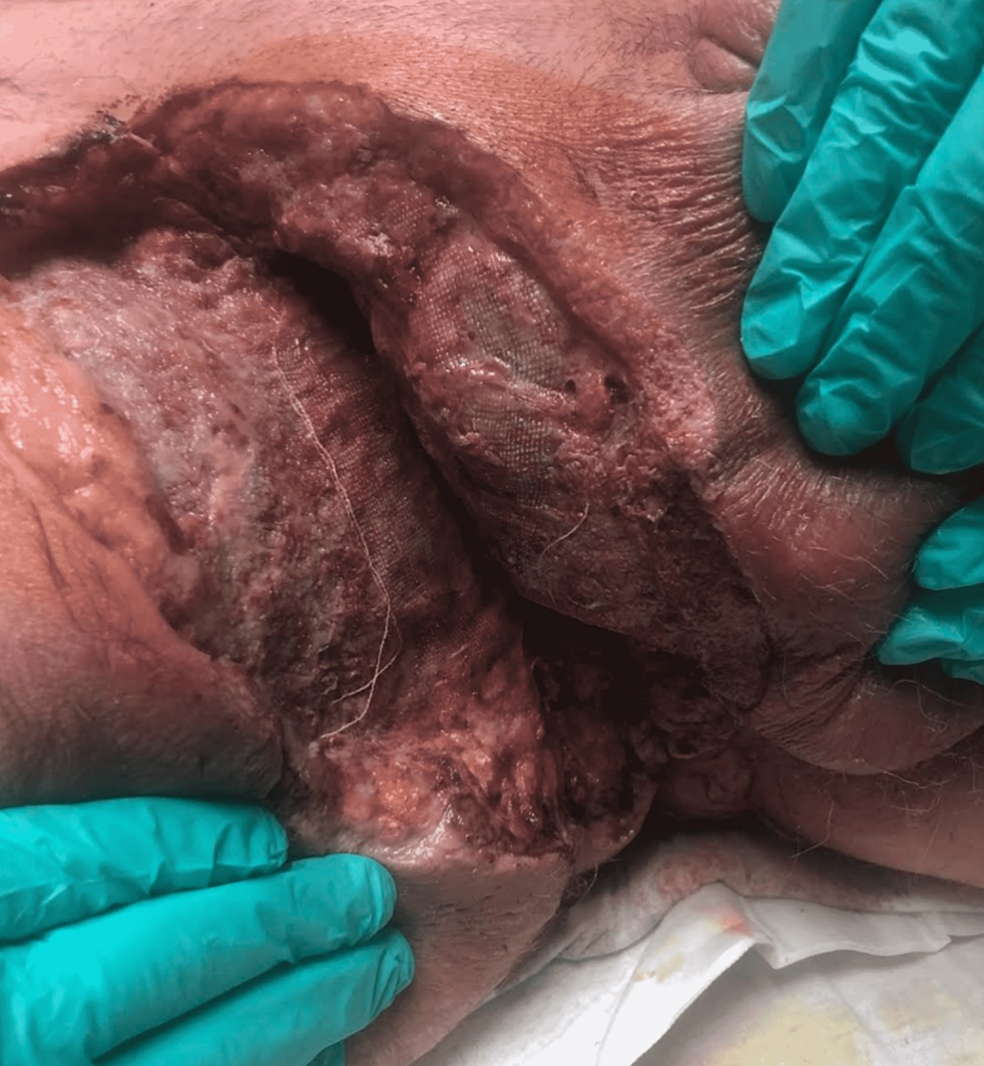
Right Inguinal region during debridement

Urology was consulted and they performed emergent soft tissue debridement of the groin, scrotum, and perineum (Figure 2).

**Figure 3:**
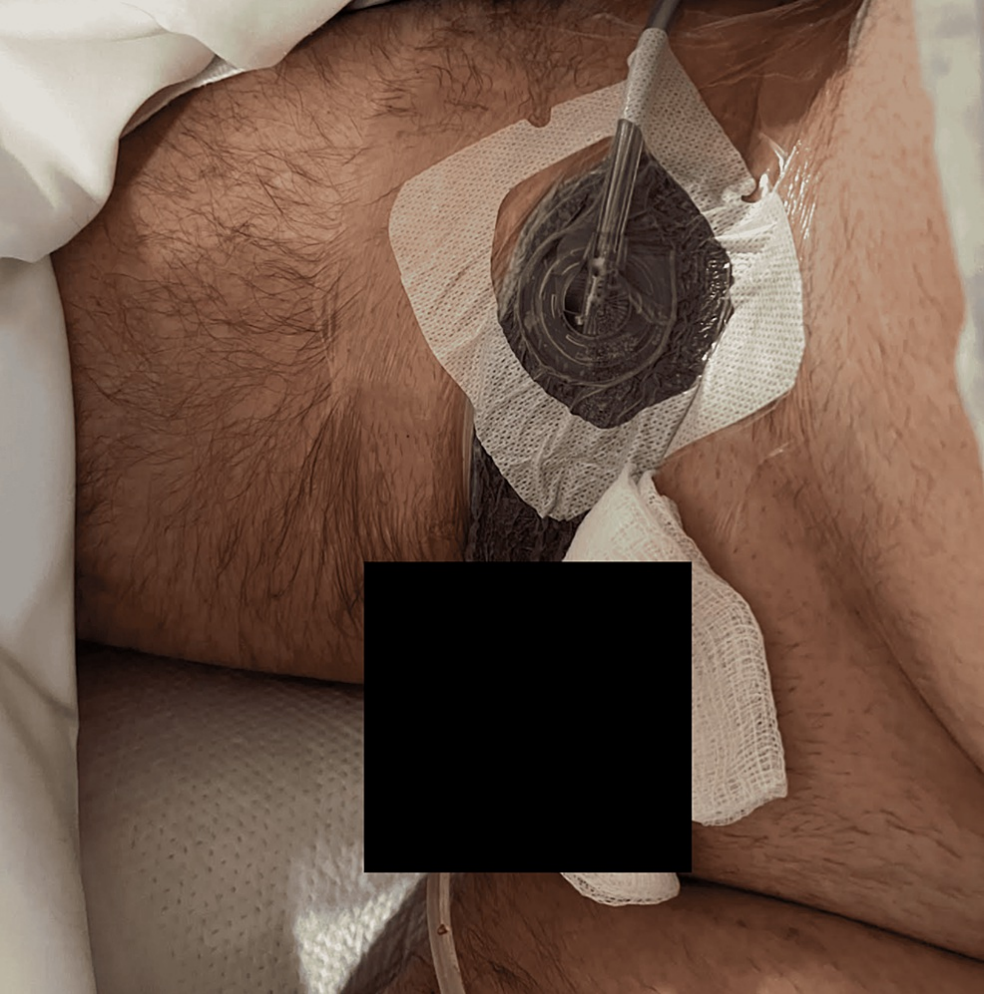
Status post-genital reconstruction surgery with Penrose drain which was removed later in the clinic

Foul-smelling dishwater-like fluid was drained. The testes and tunica vaginalis were not involved and were spared in dissection. The patient was empirically started on vancomycin, piperacillin-tazobactam, and clindamycin. Wound cultures grew *Streptococcus anginosus* and *Staphylococcus epidermidis* and the antibiotic was simplified to ampicillin-sulbactam based on culture results. The patient had right inguinal wound closure surgery of the right groin 11 days later with no complications (Figure 3).

He was discharged three days later and subsequently completed a 21-day course of antibiotic therapy with oral amoxicillin-clavulanate. The Penrose drain was removed one week following discharge. The patient did experience wound dehiscence with serosanguinous drainage but subsequently healed well.

## Discussion

This case highlights the multiple intertwined risk factors for necrotizing soft tissue infection, including diabetes mellitus, HIV infection, obesity, and empagliflozin administration. Additionally, our chart review revealed that six weeks prior to the initiation of empagliflozin, the patient had presented with a fungal rash in the bilateral axillary and groin region, which had been treated with topical clotrimazole and oral ketoconazole. Empagliflozin inhibits SGLT-2, which is expressed in the proximal renal tubules. SGLT-2 promotes glucose reabsorption at the level of the proximal tubule, reducing hyperglycemia via glucosuria. A frequently described complication is fungal genital and urinary tract infections by *Candida* species likely due to glucose-induced protein expression that enhances fungal binding to epithelial cells [2]. Pooled phase III safety data of empagliflozin has revealed a higher incidence of genital infections (but not urinary tract infections) in patients receiving empagliflozin vs. placebo [3]. This same safety data describes the mild nature of these infections and the low rate of medication discontinuation as a result; however, our case highlights the possible risk of life-threatening infectious complications associated with SGLT-2 inhibitors in select patients.

Our patient also had a history of well-controlled HIV infection with good compliance on a single-tablet protease inhibitor-based ART regimen, with an undetectable viral load for several years and CD4 count >760 u/mL. Chronic HIV infection has been implicated in the development of peripheral gangrene as part of a chronic inflammatory state, contributing to endothelial cell dysfunction, hypercoagulable state, and vasculitis [4]. Additionally, increased prevalence of Fournier’s gangrene has been described in patients living with HIV [5]. Despite these findings, given multiple confounding variables, HIV infection has not been established as an independent risk factor for necrotizing soft tissue infection.

## Conclusions

Fournier's gangrene is a rapidly progressing necrotizing fasciitis involving the perineal, perianal, or genital regions and constitutes a true surgical emergency with a potentially high mortality rate. The known risk factors include immunosuppression, type 2 diabetes, localized trauma, and catheterization. We reported a case of Fournier’s gangrene in a 55-year-old man who was HIV-positive and was on an SGLT-2 inhibitor. The patient was successfully treated with surgical intervention and antibiotics. Risk factors for genital infections (like chronic HIV infection in this case) should be weighed carefully prior to the initiation of SGLT-2 inhibitors as infectious complications may be life-threatening.
